# Updating Known Distribution Models for Forecasting Climate Change Impact on Endangered Species

**DOI:** 10.1371/journal.pone.0065462

**Published:** 2013-06-28

**Authors:** Antonio-Román Muñoz, Ana Luz Márquez, Raimundo Real

**Affiliations:** 1 Biogeography, Diversity and Conservation Research Team, Dept. of Animal Biology, Faculty of Sciences, University of Malaga, Malaga, Spain; 2 Fundación Migres, N-340, Km. 96 Huerta Grande, Pelayo, Algeciras, Spain; 3 Depto. de Didáctica de las Matemáticas, de las Ciencias Sociales y de las Ciencias Experimentales, Faculty of Education Sciences, University of Malaga, Malaga, Spain; Bangor University, United Kingdom

## Abstract

To plan endangered species conservation and to design adequate management programmes, it is necessary to predict their distributional response to climate change, especially under the current situation of rapid change. However, these predictions are customarily done by relating *de novo* the distribution of the species with climatic conditions with no regard of previously available knowledge about the factors affecting the species distribution. We propose to take advantage of known species distribution models, but proceeding to update them with the variables yielded by climatic models before projecting them to the future. To exemplify our proposal, the availability of suitable habitat across Spain for the endangered Bonelli's Eagle (*Aquila fasciata*) was modelled by updating a pre-existing model based on current climate and topography to a combination of different general circulation models and Special Report on Emissions Scenarios. Our results suggested that the main threat for this endangered species would not be climate change, since all forecasting models show that its distribution will be maintained and increased in mainland Spain for all the XXI century. We remark on the importance of linking conservation biology with distribution modelling by updating existing models, frequently available for endangered species, considering all the known factors conditioning the species' distribution, instead of building new models that are based on climate change variables only.

## Introduction

At present there are evidences suggesting that climate is warming globally and fast, partially in response to the increased output of greenhouse gases. The Report of the Intergovernmental Panel on Climate Change [1] concluded that past, present and future emissions of greenhouse gases are expected to warm the global climate between 1.4 and 5.8°C by 2100, what is a projected rate of warming much larger than the observed changes during the 20th century [2], and likely without precedent during the last 10,000 years, according to palaeoclimate data [3]. These climatic changes are already altering some physical and biological systems and have already affected the distribution and population dynamics of a number of taxa across a broad range of geographical locations and habitats [4]–[9], and are expected to have even more severe consequences over the coming century [10]. Climate is one of the main determinant factors affecting the geographical range of species [4], [11]–[13], and birds, a well-studied group of organisms, may respond to climate change changing wintering areas, migration routes and breeding grounds [14], [15], undergoing changes in their phenology [16]–[21] and their local abundances [22], and also changing their overall distributions [23]–[26]. In this way, being able to anticipate the effects of climate change on the distribution of species could improve their management and conservation policy.

A frequently used method to assess the potential impact of climate change on species is to model species distributions, relating observations to a series of environmental variables [27]. However, these predictions normally do not take into account previous knowledge about the historical, geographical, ecological and human-related factors that are known to condition the species distribution, which tend to be available for endangered species [28]. On the other hand, this knowledge is difficult to incorporate into climate change models, as the variables involved in them are not the same as those produced by the climate change scenarios. To take advantage of known species distribution models, a promising approach is to update them to the variables yielded by climatic models before projecting to the future.

An explanatory model was described for the distribution of the endangered Bonelli's Eagle (*Aquila fasciata*) in Spain based on three variables: slope, mean temperature of July and mean annual precipitation [29]. Consequently, expected modifications of the temperature in July and annual precipitation due to climate change may affect the distribution of this species along this century. The most fundamental measure of the Earth's climate is surface temperature, and precipitation is also a key element of climate [30], so this explanatory model can be used to evaluate the possible effect of climate change on the distribution of this species.

According to the predictions of the different Atmosphere-Ocean General Circulation Models (AOGCMs) and Special Report on Emissions Scenarios (SRESs) of the IPCC, in Spain there will be a decrease in precipitation and an increase in temperature through the present century. The Agencia Estatal de Meteorología (AEMET) of Spain regionalized to Spain several climate change models produced by the Intergovernmental Panel on Climate Change (IPCC), but the resulting variables of mean temperature of July and mean annual precipitation for the present were numerically different (although nominally equivalent) from those used in the existing explanatory model about Bonelli's Eagle distribution in Spain [29], which derived from actual readings of meteorological data. On the other hand, the known Bonelli's Eagle distribution model cannot be transferred to the future at face value, as the correlation among the explanatory variables is different from that existing among the AEMET variables, which affect the parameterization process and, consequently, the value of the parameters in the model. Therefore, the explanatory model needs to be updated to the AEMET variables before being fit for transference to the future scenarios.

In the present study, we modelled the future potential distribution of Bonelli's Eagle in Spain under several future climatic scenarios by updating the existing distribution model involving both climate and topography. Our aim was also to evaluate the effect of climate in relation with topography in the updated model, which could either inflate or obscure the pure effect of climate on the distribution of this cliff-nesting species, before projecting the models to the future.

## Methods

### Study area

The study area is mainland Spain, an area of 493,518 km^2^ characterized by a heterogeneous climate, which makes it particularly appropriate for analyzing different climate change scenarios. There is a mainly eastward and southward decreasing gradient of precipitation and a mainly northward-decreasing gradient of temperature [31]. Annual precipitation varies from less than 200 mm to more than 2000 mm, whereas mean annual temperatures vary from less than 6°C to more than 18°C.

Peninsular Spain has important mountain ranges, which reach a maximum altitude of 3478 m, many of them in the coastal areas contributing to isolate the central plateau from sea influences. Mainland Spain may be divided into three climatic areas: Atlantic, Mediterranean and Interior. Mild winters are found in the Atlantic area, together with cool summers, and the precipitation is abundant and regular. The Mediterranean part is characterized by hot summers and mild winters; rainfall rarely exceeds 500 mm annually and occurs mainly during spring and autumn. In the Interior, the temperatures are high in summer and low in winter, and precipitation is irregular and scarce [32].

### Target species

Bonelli's Eagle is one of the rarest raptors in Europe and is now listed as endangered [33]–[34]. During the 70 s and 80 s European populations of the species suffered a severe population decline of 20–50% [35]–[37], although in recent years the population appears to have stabilized [38], with a current estimated population of 920–1100 pairs [33]. Because of this, it is a priority-target species for conservation in Europe (Council Directive 79/409/EEC). The majority of the European population (aprox. 80%) is concentrated in the Iberian Peninsula, where this raptor has experienced a population decline of 50% over the last three eagle generations [39]. Consequently the Bonelli's Eagle is also a priority-target species for special conservation measures in Spain (Real Decreto 439/1990). Main factors involved in the decline were primarily a high mortality rate in adults and sub-adults [40], [41], and the loss of suitable habitat caused by alterations in land-use [34], [42]. Interspecific competition with other raptors for breeding sites and home-ranges could also have had an effect [43], [44].

Bonelli's eagle is a long-lived, with deferred maturity and sedentary species, with adult birds typically tied to a specific territory throughout the year [34]. Young eagles normally settle in dispersal areas during the period preceding sexual maturity that are clearly separated from the breeding range [34], [45]. The home-range size for Bonelli's eagles in the study area normally varies from 20 to 110 km^2^ [39]. Its distribution ranges from India and Southern China to the Iberian Peninsula and NW Africa [46]. In the western limit of its distribution area it occupies mainly the Mediterranean area, which is considered highly responsive to climate change because of its geographical situation between the temperate central Europe and the arid northern Africa [47], [48]. Suitable areas for this species are mountainous with a Mediterranean climate, characterized by hot summers and low precipitation [29], although human disturbance may also affect at a local scale [49], [50]. We obtained presence and absence data for a UTM of 10×10 km (5167 squares) from the last national survey conducted in 2005, which was produced with high accuracy and completeness [38].

### Updating the known species distribution model

To forecast species distributions it is necessary first to balance the impact of climate change against the effects promoted by other influential factors [13]. The ideal way to balance these different effects is to consider actual climatic data, if available, rather than fictitious climatic variables derived from AOGCM-SRES combinations, together with other drivers of species distribution. This assessment was actually done for Bonelli's Eagle in mainland Spain [29], and yielded a parsimonious model including climate and topography as main drivers of the species distribution. This model, however, is not directly transferable to the future using climate change scenarios, as the future climatic variables reflect a simulated variation of climatic conditions in relation to the modeled present climate rather than the actual present climate. The best approach in this situation is to take advantage of the known model by updating it to the simulated climate provided by the AOGCM-SRES combinations. Updating methods are re-calibration procedures that have been used to adjust previously developed models to contemporary and/or local circumstances when a new sample is available [51].

The original explanatory model [29] was updated for each combination of AOGCM and SRES, by performing the updating method 4 used in [52] -corresponding to the updating method 5 of [51]-. Consequently, we fitted a new logistic regression of the most recent distribution data published in [38] on the invariant slope *(Slop)*, and the projected mean July temperature *(Tjul)* and mean annual precipitations *(Prec)* for the period 1961–1990 by the AOGCM-SRES combination, re-estimating all the coefficients. From these logistic regressions we obtained the corresponding updated favourability functions, which represented the present updated favourability (*F_p_*) for the species in each cell,
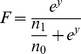
where *F* is the logit link of the favourability function, *e* is the Neperian number, *y* is the logistic regression model equation, and *n_1_* and *n_0_* are the numbers of presences and absences, respectively [53].

Some authors argue in favor of using only climatic variables in this type of models [54], given that climate is strongly correlated with topography. However, at least for mountain species, topography is an influential factor in the species distribution, not a mere surrogate of climate. We have included topography to better understand the relationship of habitat structure with the potential distribution of the species, which may balance the impact of climate change against the inertia induced by other not changing influential factor. This is especially important when dealing with a species intimately linked to cliffs. It is already known that the true effect of topography is obscured by climate in the case of Bonelli's Eagle [29], and other mountain species [13]. In the case of Golden Eagle (*Aquila chrysaetos*), another cliff-nesting raptor, topographic variables are involved in those models better explaining its occurrence [55].

To assess the extent to which climatic and non-climatic variables explain the species distribution we differentiated, in each updated favourability model, the contribution of the climatic variables from that of slope using a variation partitioning procedure, following the approach described in [29], [56]. In this way, we distinguished the Pure Climatic Factor (PCF, measured with R^2^_pClim_), i.e., the pure effect of climate on the model variation not affected by the collinearity with slope; the Pure Non-Climatic Factor (PNCF, measured with R^2^_pNClim_), i.e., the variation in the model that was due to the pure effect of slope not affected by the collinearity with precipitation and temperature; and the Shared Climatic Factor (SCF, measured with R^2^_ClimNClim_), i.e., the proportion which was assignable to their shared effect [57]–[61]. The part of the variation in the model explained by each factor (i.e. R^2^_Clim_, R^2^_NClim_) was obtained by a linear regression of the logit function of the model with the variables of each factor. Then, the pure effect of each factor was assessed by subtracting from 1 the variation of the model explained by the other factor (R^2^_pClim_ = 1 - R^2^_NClim_; R^2^_pNClim_ = 1 - R^2^_Clim_; and R^2^_ClimNClim_ = 1 - R^2^_pClim_ - R^2^_pNClim_). We also estimated the proportion of the climatic factor represented by the pure climate (ρ) for each climatic model (ρ = R^2^_pClim_/R^2^_Clim_).

The updated favourability models were projected to the future by replacing the values of *Tjul* and *Prec* by their corresponding values in the periods 2011–2040, 2041–2070 and 2071–2100 while maintaining the coefficients and the values of *Slop* (Table 1), which will not change substantially in the near future. The digital slope (*Slop*) was obtained according to the method described in [29], [62], and the climatic variables were obtained from data supplied by the Agencia Estatal de Meteorología (AEMET) of Spain and digitalized using the method explained by [63]. These data resulted from the regionalization to Spain of the climate change models produced by the Intergovernmental Panel on Climate Change (IPCC).

**Table 1:** Variables used to model the species distribution.

Code	Variables
***Slop***	Slope (°) (calculated from altitude)(1)
***Prec***	Annual precipitation (mm)(2)
***TJul***	July mean temperature(2)

(1) US Geological Survey (GTOPO30) (http://edcdaac.usgs.gov/gtopo30/gtopo30.asp);

(2) Agencia Estatal de Meteorología of Spain (AEMET), Ministerio de Medio Ambiente (http://www.aemet.es/es/elclima/cambio_climat/escenarios).

### Climate change scenarios

We used four different AOGCMs: CGCM2 from the Canadian Climate Centre for Modeling and Analysis, ECHAM4 from the Max Planck Institut für Meteorologie, and HadAM3H and HadCM2SUL from the Hadley Centre (U.K.), which differ in horizontal and vertical resolutions and in the parameterizations of physical processes (convection, land surface processes, cloud cover, and radiation, among others). According to the data obtained from the AEMET the circulation models CGCM2 and ECHAM4 were run with the conditions forecasted by the SRES A2 and B2 [64], HadAM3H was run with the scenario A2, and HadCM2SUL was run with the scenario IS92a, as they are the scenarios regionalized for Spain [65] (See Table 2). Scenarios A2 and B2 represent an intermediate position of the range of projected temperature change scenarios for Spain, A2 being medium-high and B2 medium-low [66]. The A2 storyline describes a very heterogeneous world with a regionally oriented economic development preserving local identities, and assumes modest reductions in overall population growth. The B2 storyline describes a world in which the emphasis is on environmental sustainability and local solutions to economic and social issues, and assumes more substantial reductions in overall population growth.

**Table 2:** The combination of AOGCMs and scenarios used in this study.

	SRE		
AOGCM	A2	B2	IS92a
CGCM2	x	x	
ECHAM4	x	x	
HAdAM3	x		
HadCM2SUL			x

All the climatic models were run for the periods: 1961–1990, 2011–2040, 2041–2070, 2071–2100, with the exception of the HadAM3 which only had data for 1961–1990 and 2071–2100 (Table 2), obtaining in each cell a value of expected future favourability (*F_f_*) according to each AOGCM-SRES combination.

Applying the expression 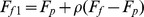 we calculated the minimum or the maximum climatic effect over the species distribution, i.e., *F_f_* and *F_f_*_1_ represent the limits of the forecasted effects of climate change on the spatial distribution of the favourability for the species.

As favourability values may be interpreted as the degree of membership of the sites to the fuzzy set of localities favourable to the species [53], [63], we used some fuzzy logic operations [67] to calculate, for each future projection, the IOMS features of the forecasted effect of climate change of the species favourability proposed by [63], namely the increment in favourability (I), the favourability overlap (O), the favourability maintenance (M), and the forecasted shift in favourability (S) with respect to the 1961–1990 period:
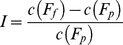


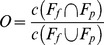


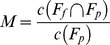


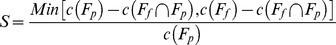
where,

*c(X)* is the cardinality of the *X* fuzzy set, that is, the sum of all cells' membership degrees to the fuzzy set *X*.
*F_f_* is the fuzzy set of future favourable areas for the species, and the membership degree of each cell to *F_f_* is defined by the future favourability value for the species in the cell.
*F_p_* is the fuzzy set of present favourable areas for the species, and the membership degree of each cell to *F_p_* is defined by the present favourability value for the species in the cell.

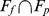
 is the intersection between future and present favourabilities, and the membership degree of each cell to 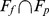 is defined by the minimum of the two favourability value for the species in the cell.

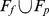
 is the union between future and present favourabilities, and the membership degree of each cell to 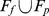 is defined by the maximum of the two favourability values for the species in the cell.

We proceeded analogously for obtaining the IOMS features comparing *F_p_* with *F_f1_*.

Positive values of increment (*I*) indicate a favourability expansion for the species, that is, a gain of favourable areas, whereas negative values of *I* mean a net loss of favourability areas for the species. High values of overlap (O) indicate that the distributions of future local favourability values are predicted to be similar to that shown at present. Maintenance (M) indicates the degree to which current local favourability values are predicted to persist in the future, so that low values of M are of more conservation concern that high M values. Favourability shift (S) measure the proportion of the present favourability that is predicted to be lost in the future but may be compensated with new favourability opportunities elsewhere.

## Results

Coefficients of the logit function of the favourability models for the period 1961–1990 are shown in Table 3. Table 4 shows the results of the variation partitioning of the favourability model, specifying the percentages of variation explained by the Pure Non-Climatic Factor (PNCF), the Pure Climatic Factor (PCF), the interaction that is due to Share Climatic Factor (SCF) and the proportion of pure climatic factor in relation to the whole climatic factor (ρ). In all favourability models climate had a more important effect than topography on the distribution of the species. All SCF values were negative, which indicates that topography tends to obscure the effect of climate on the species distribution.

**Table 3:** Coefficients in the logit function (*y*) of the favourability models for the period 1961–1990.

AOGCM	*y*
**CGCM2-A2**	0.319 * *Slop* - 0.0023 * *Prec*+0.366 * *TJul* - 9.51
**CGCM2-B2**	0.319 * *Slop* - 0.0023 * *Prec*+0.366 * *TJul* - 9.51
**ECHAM4-A2**	0.375 * *Slop* - 0.0035 * *Prec*+0.428 * *TJul* - 12.25
**ECHAM4-B2**	0.375 * *Slop* - 0.0035 * *Prec*+0.428 * *TJul* - 12.25
**HAdAM3-A2**	0.348 * *Slop* - 0.0043 * *Prec*+0.296 * *TJul* - 8.29
**HadCM2-IS92a**	0.312 * *Slop* - 0.0017 * *Prec*+0.394 * *TJul* - 10.18

For each AOGCM *Prec* and *TJul* are the forecasted for them.

**Table 4:** Results of the variation partitioning of combined favourability model.

	CGCM2		ECHAM4	HadAM3H	HadCM2SUL
	A2	B2	A2/B2	A2	IS92a
PNCF	30.6	30.6	32.1	33.1	31.4
PCF	92	92	94.7	93.5	90.3
SCF	−22.6	−22.6	−26.8	−26.6	−21.7
ρ	1.326	1.326	1.395	1.398	1.316

Values shown are the percentages of variation explained by the Pure Non-climatic Factor (PNCF), the Pure Climatic Factor (PCF) and the interaction that is the Share Climatic Factor (SCF). (ρ).: Proportion of pure climatic factor in relation to whole climatic factor.

**Figure 1:**
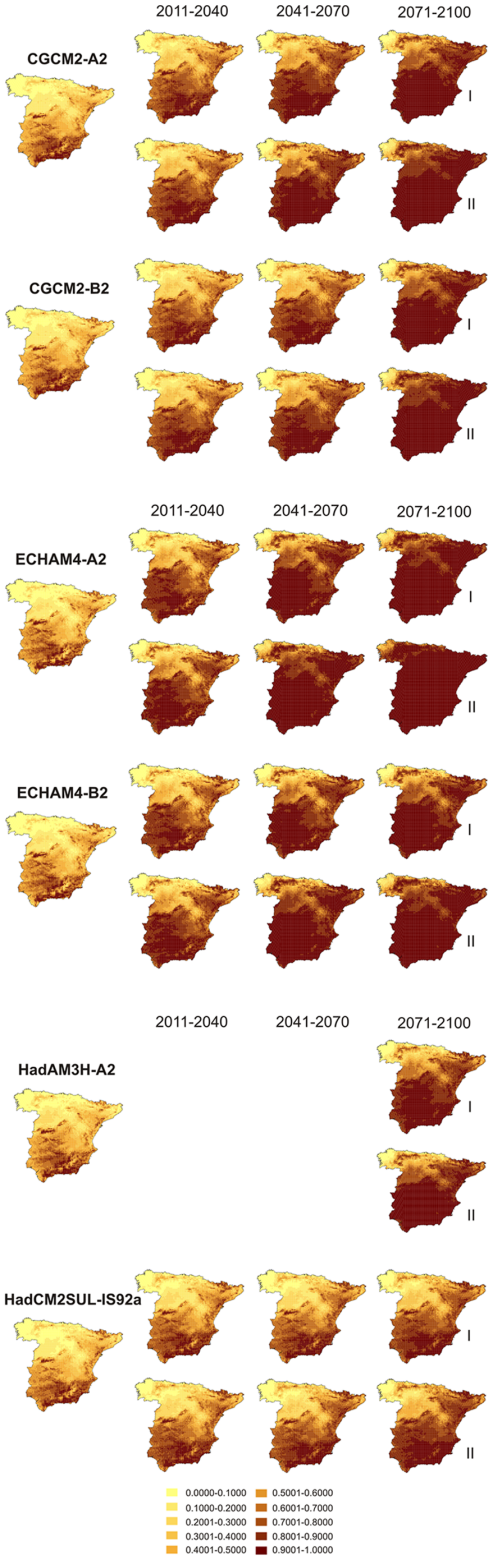
Favourability values forecasted at each 10 km×10 km UTM square of mainland Spain for Bonelli's Eagle, according to each climatic model and for each considered period. I and II indicate the minimum and maximum expected change in favourability, respectively.

Figure 1 shows the future favourability for *A. fasciata* according to the climatic conditions forecasted for every time period by each AOGCM and SRES combination, including the minimum and maximum expected change in favourability for every case. Minimum and maximum values of increment (*I*), overlap (*O*), maintenance (*M*), and shift (*S*) in favourability between the 1961–1990 period and the forecasted future favourability are shown in Table 5. All the climatic models forecasted the maintenance of Bonelli's Eagle present favourability areas, as well as its expansion (positive increment) during all the XXI century and especially in the last period (2071–2100).

**Table 5:** Values of the rates of increment (I), overlap (O), maintenance (M) and shifting (S) of favourability forecasted for each future projection with respect to the 1961–1990 period.

			I		O		M		S		CFf	
			*I*	*II*	*I*	*II*	*I*	*II*	*I*	*II*	*I*	*II*
**CGCM2**	**A2**	**2011–2040**	0,392	0,512	1,392	1,512	1,0	1,0	0,0	0,0	2869,0	3116,1
		**2041–2070**	0,716	0,899	1,716	1,899	1,0	1,0	0,0	0,0	3536,8	3914,3
		**2071–2100**	0,995	1,182	1,995	2,182	1,0	1,0	0,0	0,0	4113,0	4497,5
	**B2**	**2011–2040**	0,362	0,472	1,362	1,472	1,0	1,0	0,0	0,0	2811,0	3038,2
		**2041–2070**	0,560	0,719	1,560	1,719	1,0	1,0	0,0	0,0	3220,8	3548,9
		**2071–2100**	1,010	1,194	2,010	2,194	1,0	1,0	0,0	0,0	4149,6	4528,8
**ECHAM4**	**A2**	**2011–2040**	0,529	0,714	1,529	1,714	1,0	1,0	0,0	0,0	3143,3	3524,2
		**2041–2070**	0,936	1,177	1,936	2,177	1,0	1,0	0,0	0,0	3980,2	4476,5
		**2071–2100**	1,213	1,399	2,213	2,399	1,0	1,0	0,0	0,0	4551,0	4933,0
	**B2**	**2011–2040**	0,485	0,656	1,485	1,656	1,0	1,0	0,0	0,0	3053,1	3406,1
		**2041–2070**	0,817	1,053	1,817	2,053	1,0	1,0	0,0	0,0	3735,4	4221,9
		**2071–2100**	0,966	1,200	1,966	2,200	1,0	1,0	0,0	0,0	4042,8	4524,8
**HadAM3**	**A2**	**2011–2040**	0,236	0,329	1,236	1,329	1,0	1,0	0,0	0,0	2542,5	2735,1
		**2041–2070**	0,321	0,445	1,321	1,445	1,0	1,0	0,0	0,0	2717,1	2972,3
		**2071–2100**	0,517	0,704	1,517	1,704	1,0	1,0	0,0	0,0	3121,0	3505,1
**HadCM2SUL**	**IS92a**	**2071–2100**	0,784	0,956	1,784	1,956	1,0	1,0	0,0	0,0	3679,8	4034,4

*cF_f_* is the cardinality of the fuzzy set of favourable areas forecasted for the respective future period. *I*: considering the apparent climatic effect, and *II*: considering the pure climatic effect, at present and in each future period for the four climatic models considered.

## Discussion

### The modelling approach

Generalised Linear Models (GLMs) formulate the relationship between distribution and environmental variables explicitly, and thus are appropriate tools to generate hypotheses about how species respond to spatial and environmental variability and to provide insights into the potential response to regional climate change [68]. These methods have the advantage of modelling both presence and absence data, which is critical for threatened species [69]. Although some authors recommend the use of profile modelling techniques that supposedly only require presence data, and thus are thought not to be affected by false absences [70]–[72], these methods are equally affected by missing presences (i.e., false absences) while not paying due attention to the specific causes of absences. We modelled absences explicitly because the true absence of a species from an area may be due to ecological, historical, or anthropogenic reasons, all of which are relevant factors in biogeography and conservation [56], [73], [74]. When consistent absence data are available, the explicit consideration of absences in the regression analysis improve the quality of the models, as they provide more explicit information about less favourable locations or unfavourable conditions for the species. This is why assessing the quality of the absence data (for example, measuring specificity) should be considered as important as the assessment of presence data in modelling procedures [13], [75].

### Variation partitioning

Accurate predictions about future species distributions and responses to future climate largely depend on the combination of the causal factors involved. Our models for Bonelli's Eagle included a climatic and a topographic factor, and depending on the importance of the former the future projections of the species distribution will be more or less affected by climate change. By using variation partitioning and weighting the effect of climate in relation to topography, we have evaluated the pure contribution of climate, not affected by the covariation with topography, in making a given area favourable for this mountain species. The effect of temperature and precipitation (i.e. the pure climatic factor) is obscured by slope (i.e. the non-climatic factor) in the amount expressed by the negative shared effect shown in Table 5. The pure effect of climate in the models is roughly three times that of topography (see Table 5) in all combined favourability models. This is probably the reason why the species is absent from Iberian mountains outside Mediterranean areas.

### Management in a changing climate

Over the last century, mean annual temperatures have increased by 0.8°C in Europe, at the same time as annual precipitation has increased by 10–40% in northern Europe and decreased by up to 20% in parts of southern Europe [76]. Climate change is expected to have a noticeable effect on bird populations across a variety of habitats, as both ambient temperatures and levels of precipitation have a direct influence on the distribution, survival rates and productivity of individual species, and thus on population sizes [29], [77]–[79].

Generalist species are thought to deal with rapid environmental change, while it is likely that species with more specialized ecological niches will face more severe challenges [80]. In Europe there are examples of bird species with more northerly geographic distributions that have declined, populations with more southerly distributions that have remained relatively stable or increased [22], [81], and even cases in which African species have recently colonized southern Europe [82], [83].

In the Mediterranean context Spain is a highly important area for bird conservation. It is the European member state with the largest surface area devoted to SPA (Special Protection Areas) for birds [84], and it is among the most responsive areas to global climate change due to its geographic situation [47], [48]. In general, climate change implies a challenge for the current conservation policy, which generally assumes static species ranges, and do not consider the dynamism of the reserve borders nor the natural system dynamics caused by a changing world. In the case of Bonelli's Eagle in Andalusia (South of Spain), which is one of the most important strongholds for the species in Europe, 52.4% of the breeding territories are currently in protected areas [85], but most of the new favourable areas are predicted to occur outside the network of Andalusia's reserves, and thus the percentage of “unprotected” eagles is expected to increase. In Eastern Spain it has been demonstrated that the current network of special protected areas becomes insufficient to protect Bonelli's Eagle [86]. Species are likely to change their distributions, adjusting it to the emergence of new favourable and unfavourable areas, and therefore their representation levels in static reserves are prone to be altered [87], [88]. Therefore, an effort should be made to spatially coordinate reserve management to capture these biological dynamics among multiple protected areas and across the landscape [89].

The capacity to simulate the potential changes in the distribution range of Bonelli's Eagle in Spain as precisely as possible is important to favour the conservation of the species, especially taking into account that Spain concentrates most of the European population. Changes in temperatures and precipitation patterns may have direct and indirect effects on the survival rates and productivity of the species [90], thus influencing the viability of its populations.

Our analyses indicate that the favourable areas for Bonelli's Eagle, according to all the AOGCM and scenarios used, will increase during the XXI century in Spain. The impact of climate change on this species in our study area will not be negative as it occurs for other bird species that are expected to suffer important decreases in their distribution area [26], [91]. We recommend that to model species distributions in the future, multiple climatic models, i.e. the combination of AOGCMs and SRESs, should be used.

### Predicting the future favorability and potential distribution

It is widely acknowledged that species distribution models provide a simplified representation of the processes governing the geographic distributions of species [27], [92]. Although it is difficult to fully explore uncertainties arising from the large number of AOGCMs that are currently being generated, our approach and results are consistent in predicting an increase in climatic favourability for all the scenarios used. However, the intensity of the forecasted increment in favourability differ for the AOGCMs used, ranging from the more drastic changes predicted according to ECHAM4 to the more conservative predictions of HadCM2SUL. Our impression after visual inspection of Figure 1 is that predictions of HadCM2SUL seem to be more reasonable, but this could be affected by an ill-founded expectation of moderate changes in nature.

The uncertainty associated to the differences in AOGCMs and SRESs has already been measured [63]. In this work we assessed a new source of uncertainty associated to the models, which derives from not knowing the exact role of climate in the biogeographical response of the species. At least, our approach allows putting limits to the minimum and maximum expected influence of climate on the species distribution and, consequently, forecasting minimum and maximum future changes in environmental favourability. Another possible source of uncertainty, especially in those species with a projected increase in distribution, as is our case, is the overestimation due to the truncated response curves [93]. Considering new environmental conditions that are outside of the calibration range could lead to erroneously predict the new conditions as favourable overlooking the fact that warmer temperatures and lower values of precipitation could be unsuitable for the species (e.g. physiological limitations or new conditions of competence) [54]. In our case, 99,1% of the predicted new favourable squares are within the range of the function *y*.

It is necessary to consider that an increase in the existence of favourable areas does not necessarily mean an increase in the species distribution. Human interaction will probably prevent Bonelli's Eagles from establishing in many climatically-favourable zones. Although this species may tolerate high levels of human disturbance [29], [94]–[96], the main causes of mortality for Bonelli's eagle in Spain are human induced, mostly due to power lines casualties and also direct persecution [41]. It is remarkable that a significant proportion of the new favourable areas are predicted in flat or undulating landscapes, which lack natural perching sites for the eagles and would favor the use of electric pylons, making them more vulnerable to electrocution. Therefore, in order to enhance the conservation of the species, mitigation measures to prevent power lines-induced mortality might be accordingly contemplated in these areas, considering that management actions normally require long temporal scales. In the case of the endangered Spanish Imperial Eagle (*Aquila adalberti*) it has already been demonstrated that eagle electrocution is an affordable problem whenever there is political interest and financial support [97].

Since Bonelli's Eagle is a species of conservation concern in Europe and Spain, we take advantage of the pros provided by a regional pre-existing distribution model, and the most recent distribution data, together with the simulated climate change variables. This could be of particular interest to the species in the European context because mainland Spain includes approximately 80% of European Bonelli's Eagles [33].

This paper predicts an increase in environmental favourability for the species in the Iberian Peninsula, but many of these new favourable areas are outside mountain ranges and have little or no availability of cliffs, which currently are the usual nesting areas. In Spain Bonelli's Eagle breeds mainly in rocky substrates, since 95.5% of the nests are found in this substrate, while trees and power lines are occasionally used, 4% and 0.5% respectively. Our models can be considered as realistic only if the nesting behaviour of the species in Spain changes significantly to use trees or power lines much more than currently. On a global scale Bonelli's eagle occupies mountains, cliffs, crags, gorges, hills and plains with forest or woodland [98], [99], although in some areas built their nest on lofty trees, as in southern India [99] and Portugal. In the case of neighboring Portugal the proportion of pairs nesting on trees is completely different from that found in Spain. There 64% of the population nest in trees [100], like Cork Oaks, Pines and large Eucalyptus, and in the south of the country 61 out of the 65 pairs (94%) are tree-nesters. This demonstrates the plasticity of the species to choose nesting substrate and to breed in trees in those favorable regions with no mountains, which could mean a future increase in the range of Bonelli's Eagle if it starts to breed also in trees in Spain. Interestingly, the African Hawk-eagle (*Aquila spilogaster*), the sister species of Bonelli's Eagle, is distributed in tropical Africa south of the Sahara, lives in woodlands and breeds exclusively on trees, mainly in *Acacia* riparian woodland, *Baikiaea* and mixed woodland [101].

Species may respond to global climate change by shifting their geographical distribution in absence of any evolutionary change [5] but, as it has been already pointed out, evolutionary adaptation can be rapid helping species achieve new ecological opportunities arising from climate change [102]. Another source of uncertainty when projecting species distribution models to the future is our inability to forecast how species might express phenotypic plasticity to changing environmental conditions [103]. For this reason, to consider the evolutionary potential of species and including the possibility of evolution in distribution modelling would provide detailed information of great interest in order to better determine the effect of climate change on species. It would also allow incorporating this information into better informed management programs designed to prevent biodiversity loss under rapid climate change. Long-term monitoring and observations, especially in long-lived territorial raptors characterised by deferred maturity [104], and future updating of pre-existing models, considering new distribution ranges known in the future and also new variables, are needed to provide an assessment of the predictions about climate change and about Bonelli's Eagle response by the possible changing of nesting behaviour and increase of its distribution range.

In general, global distribution models are preferable to regional models, as predicting the future distribution of a species from a part of its range could be oblivious to the variation in climate tolerance that is not present in the studied area. We updated a regional distribution model because this was the only pre-existing model available. Nevertheless, if a global niche model of the species were at hand, we would recommend it to be updated to the target region anyway, because in a global model the relationship between climate and non-climatic factors, and their separate and combined effects on the species distribution, are averaged throughout the species range, in this case from Portugal to China, including Africa and Indonesia, while some factors may be more, or less, critical than average in specific zones of the species range. The territory we analyzed in this study is characterized by a heterogeneous climate and hosts the core of Bonelli's Eagle European population, which makes it particularly appropriate for analyzing different climate change scenarios. Additionally, mainland Spain encompasses the whole variability of breeding behaviours known for the species in its entire range. Another possible advantage of focusing the study of the effect of climate change on a specific and discrete part of its distribution area is that allopatric distribution of Bonelli's Eagle (e.g. China, the Indian subcontinent and Indonesian populations), probably represent relatively different natural histories, and presumably different responses to environmental conditions. Thus, the updating of pre-existing models allows retaining the potential of these models, either regional or global, while recalibrating them to optimize their performance in specific situations.

### Conclusions

To perform good species distribution models is time consuming. When working with many species simultaneously modelling may become a routine task which does not allow paying the necessary attention to the uncertainty related to each species. In this article we showed the value of using already existing models in well studied species to forecast climate change impacts, remarking the importance of linking conservation biology with distribution modelling by updating existing models, since conservation objectives are more likely to be achieved when knowledge informs actions. Models of this kind are scarce, but they are sometimes available for species of conservation concern and it is preferable to update them considering all the known factors conditioning the species' distribution to better infer climate change effects, instead of building new models that are based on climate change variables only.
